# Anticancer Effects of Five Biflavonoids from *Ginkgo Biloba* L. Male Flowers In Vitro

**DOI:** 10.3390/molecules24081496

**Published:** 2019-04-16

**Authors:** Min Li, Bin Li, Zi-Ming Xia, Ying Tian, Dan Zhang, Wen-Jing Rui, Jun-Xing Dong, Feng-Jun Xiao

**Affiliations:** 1 Beijing Institute of Radiation Medicine, Beijing 100850, China; limin82057@163.com (M.L.); jkylibin@hotmail.com (B.L.); naisixx@163.com (Z.-M.X.); hq6106@aliyun.com (Y.T.); danzhang01@foxmail.com (D.Z.); 18232627383@163.com(W.-J.R.); 2 School of Nursing, Jilin University, Changchun 130012, China

**Keywords:** *Ginkgo biloba* flowers, biflavonoids, bilobetin, isoginkgetin, anticancer

## Abstract

*Ginkgo biloba* L., an ancient dioecious gymnosperm, is now cultivated worldwide for landscaping and medical purposes. A novel biflavonoid—amentoflavone 7′′-*O*-β-d-glucopyranoside (**1**)—and four known biflavonoids were isolated and identified from the male flowers of *Ginkgo*. The anti-proliferative activities of five biflavonoids were evaluated on different cancer lines. Bilobetin (**3**) and isoginkgetin (**4**) exhibited better anti-proliferative activities on different cancer lines. Their effects were found to be cell-specific and in a dose and time dependent manner for the most sensitive HeLa cells. The significant morphological changes validated their anticancer effects in a dose-dependent manner. They were capable of arresting the G2/M phase of the cell cycle, inducing the apoptosis of HeLa cells dose-dependently and activating the proapoptotic protein Bax and the executor caspase-3. Bilobetin (**3**) could also inhibit the antiapoptotic protein Bcl-2. These might be the mechanism underlying their anti-proliferation. In short, bilobetin (**3**) and isoginkgetin (**4**) might be the early lead compounds for new anticancer agents.

## 1. Introduction

Ranking second only to cardiovascular disease, cancer is a leading cause of death globally [1]. Conventional surgery, radiotherapy, and chemotherapy are three primary cancer treatments [2]. Among them, chemotherapy is a useful and significant therapy for cancer patients in clinical applications [3]. At least 60% of approved anti-cancer drugs have natural product sources [4,5]. Natural compounds often become the lead compounds of clinical anticancer drug candidates [6]. Therefore, searching for lead compounds from natural products in cancer therapy remains desirable in modern anticancer drug discovery [5].

*Ginkgo biloba* L., has been used as a Chinese herb for thousands of years against asthma and bronchitis [7]. Modern pharmacological studies revealed its antioxidant, anti-inflammatory [8], neuroprotective effects [9], its improvement of cardiovascular and peripheral vascular disorders, as well as the antiplatelet aggregative activity [10] of *G. biloba* leaf extract (EGb). Its main bioactive constituents have been demonstrated as flavonol glycosides and terpene trilactones [11]. Furthermore, previous studies have shown that both *G. biloba* leaf extract (EGB) and exocarp extract (GBEE) possess inhibitory effects on various cancer cells [12,13]. Some monomeric compounds were also reported to have anti-proliferation activity on different cancer cells, including ginkgolic acid [14], Ginkgolide B [15], and several biflavonoids [16,17,18]. 

The gymnosperm *Ginkgo* is dioecious and its male flowers with catkin blossom from late March to the middle of April for only three to seven days, varying in different areas of China [19]. So far, there is little correlative literature illustrating the exact chemical constituents and bioactivities of the flowers. In our previous study, we isolated 18 compounds including 4 biflavonoids from *Ginkgo* male flowers and reported their anti-inflammatory activities in LPS-induced RAW 264.7 cells [20]. In our continuing efforts to investigate the constituents and bioactivities of *G. biloba* flowers, a novel biflavonoid as well as four known ones were isolated and evaluated for anti-proliferation activities on three kinds of cancer cells for the first time. Then, the active biflavonoids were further tested in five more cancer cells to select the most sensitive cells. We also preliminarily clarified the underlying anti-proliferative mechanism of the active biflavonoids in the most sensitive cancer cells.

## 2. Results

### 2.1. Structure Elucidation

**Figure 1:**
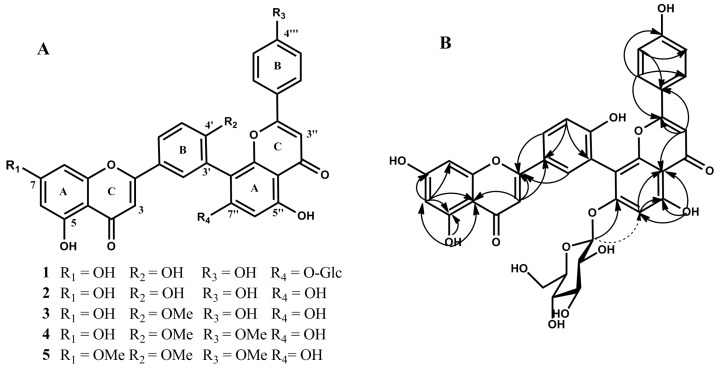
Structures of compounds **1**–**5** (**A**). Key heteronuclear multiple bond correlation (HMBC) correlations (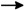) and nuclear overhauser enhancement spectroscopy (NOESY)correlations (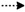) of compound **1** (**B**).

The CHCl_3_- and EtOAc-soluble fractions were all subjected to column chromatography repeatedly to investigate their chemical constitutions, which afforded a novel biflavonoid (**1**) and four known biflavonoids (**2**–**5**) (Figure 1A).

Compound **1** was isolated as a yellow amorphous powder. The molecular formula was identified as C_36_H_28_O_15_ by the high resolution electronspray ionization mass spectrometry (HR-ESI-MS) 701.1505 [M + H]^+^, corresponding to twenty-three degrees of unsaturation. The ^1^H nuclear magnetic resonance (^1^H-NMR) spectra signals revealed the existence of five hydroxyl groups and twelve aromatic protons, indicating that compound **1** was a biflavone. The ^1^H- and ^13^C-NMR chemical shifts of compound **1** were closely similar to those of compound **2** except for the presence of an additional sugar moiety and preliminarily inferred that the mother nucleus of compound **1** was compound **2** (amentoflavone). The proton chemical shifts of δ_H_ 7.60 (2H, d, *J* =8.8 Hz), δ_H_ 6.72 (2H, d, *J* = 8.8 Hz) showed a set of AA'BB' spin system of the B ring. In another B-ring, ortho and meta proton coupling pattern revealed an ABX spin system. Compared to the C-7″ of amentoflavone (δ_C_ 161.9) [21], the upfield shift of C-7″ (δ_C_ 160.45) showed that the sugar moiety was attached to C-7″. The heteronuclear multiple bond correlation (HMBC) experiments demonstrated that the anomeric proton signal at δ_H_ 5.18 (d, *J* = 7.7 Hz, 1H) is correlated with the C-7″ (δ_C_ 160.45). The nuclear overhauser enhancement spectroscopy (NOESY) experiments reconfirmed the location of the sugar moiety for the spatial correlation between the anomeric proton signal (δ_H_ 5.18) and the aromatic proton H-6″ (δ_H_ 6.79) (Figure 1B). Acid hydrolysis of compound **1** yielded a d-glucose which was identified by comparison with authentic samples on thin layer chromatography (TLC). The anomeric proton signal at δ_H_ 5.18 (d, *J* = 7.7 Hz, 1H) indicated the β-configuration for the glucopyranosyl moiety. Detailed analysis of heteronuclear singular quantum coherence (HSQC) and HMBC correlations clearly elucidated the structure of compound **1** as amentoflavone 7″-*O*-β-d-glucopyranoside.

The identification of the four known biflavonoids, including amentoflavone (**2**) [22], bilobetin (**3**), isoginkgetin (**4**) [21], sciadopitysin (**5**) [23], were reported in our previous studies [20].

### 2.2. Cytotoxic Activity Evaluation

The cytotoxic activities of five biflavonoids were evaluated on HepG2, HeLa, and NCI-H460 cell lines after treatment of different biflavonoids from 3 μM to 100 μM for 48 h. The results are expressed as half maximal inhibitory concentration (IC_50_) values in Table 1. Compound **3** showed anti-proliferative activities only on HeLa and NCI-H460 cell lines with IC_50_ values of 36.42 and 14.79 μM. Compound **4** exhibited different degrees of cytotoxic activities in all three cancer cells with the IC_50_ from 8.38 to 42.95 μM. However, compounds **1**, **2**, and **5** did not exhibit any anti-proliferative activities on all three cells. So, the active biflavonoids, compounds **3** and **4**, were further tested in five more cancer cells to select the most sensitive cells. As shown in Table 2, for compound **3**, the IC_50_ value for different tumor cells was observed in an order of HeLa < Daudi < NCI-H460 < K562 < MCF-7 < SKOV3 < MIAPaca-2 < HepG2. For compound **4**, the order of IC_50_ value for different tumor cells was HeLa < K562 < Daudi < HepG2< SKOV3 < NCI-H460 < MIAPaca-2 < MCF-7. Thus, the inhibitory effects of compounds **3** and **4** on the proliferation of tumor cells are cell type dependent and the human cervical carcinoma cell line is the most sensitive cells for both compounds **3** and **4** with the IC_50_ of 14.79 μM and 8.38 μM, respectively. 

**Figure 2:**
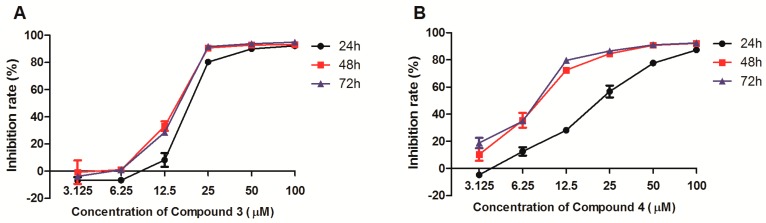
Effects of compounds **3** (**A**) and **4** (**B**) on viability of HeLa cell at different concentrations for different incubation time. HeLa cells were incubated with compound **3** or **4** at the concentration of 3.1 μM to 100 μM, and then the cytotoxic activity was evaluated using the 3-(4,5-dimethylthiazol-2-yl)-2,5-diphenyltetrazolium bromide (MTT) assay at 24 h, 48 h, and 72 h, respectively. The values are expressed as mean ± SD of triplicate experiments.

To further assess the time-dependent effect of compound **3** and **4** on cytotoxicity, HeLa cells were incubated with compound **3** or **4** at the concentration of 3 μM to 100 μM for different periods of time. As shown in Figure 2, over time, the inhibition rate of compound **3** on HeLa cells takes on roughly increasing tendency with the IC_50_ of 19.65 ± 1.4, 14.79 ± 0.64, and 15.17 ± 0.26 μM. When the concentration of compound **3** was greater than 6.25 μM, the anti-proliferative activities of compound **3** were enhanced dose-dependently. With the increasing treated time, the inhibitory effects of compound **4** were strengthened in a dose-dependent manner with the 22.54 ± 2.16, 8.38 ± 0.63, and 7.56 ± 0.21 μM for **4**, respectively. 

### 2.3. Morphological Changes

**Figure 3:**
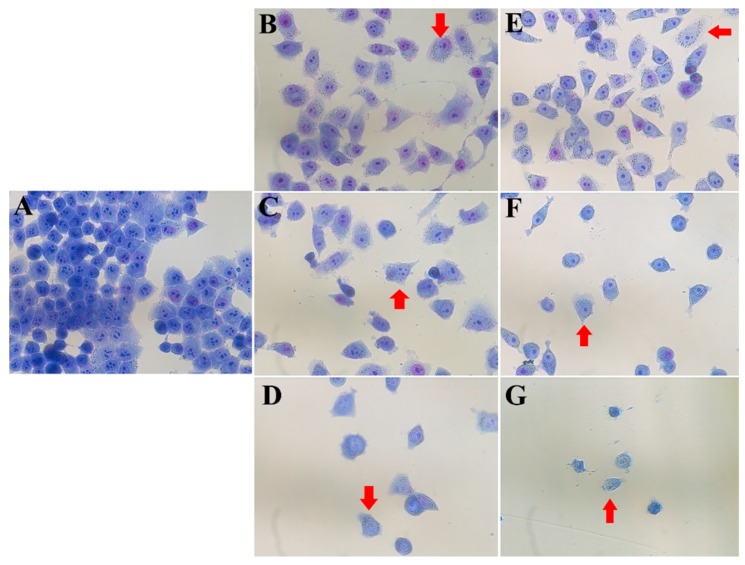
Morphological structure of HeLa cancer cells through Giemsa staining after 48 h of treatment with different concentration of compounds **3** or **4**. (**A**) control (untreated), (**B**) compound **3** (15 μM), (**C**) compound **3** (20 μM), (**D**) compound **3** (25 μM), (**E**) compound **4** (10 μM), (**F**) compound **4** (15 μM), and (**G**) compound **4** (20 μM). The arrows show the obviously changed cells.

To assess whether compounds **3** and **4** can induce any morphological changes, HeLa cells were incubated with different concentrations of compound **3** or **4** and stained with Giemsa. Then cells were observed and photographed using a microscope. Figure 3 revealed that both compounds **3** and **4** can considerably change the cellular morphology dependent on the concentration. The HeLa cells in the control group (untreated) exhibited a regular appearance, intensive growth, integral cell membrane, clear nuclear membrane and nucleolus (Figure 3A). HeLa cells appeared to be vacuolated in Figure 3B,E. Fragmented nuclear membranes and nuclear condensation can be observed in Figure 3C,F. Then, the cells were destroyed and regular nuclei were hardly found, as seen in Figure 3D,G.

### 2.4. Cell Cycle Analysis

**Figure 4:**
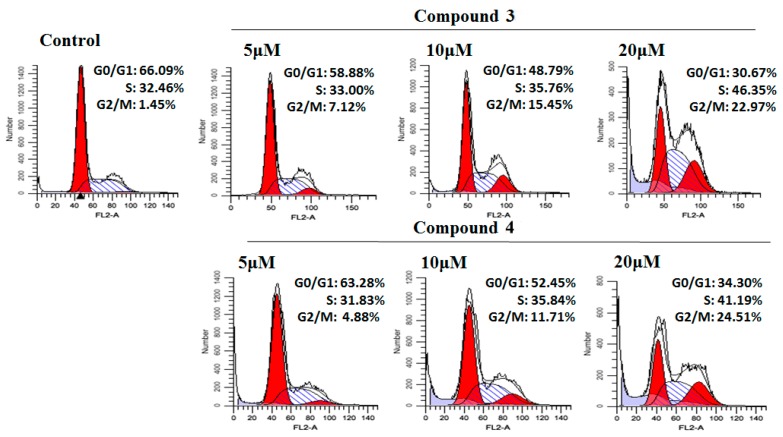
Cell cycle analysis of HeLa cells treated with compounds **3** and **4** (5 μM, 10 μM, and 20 μM) for 24 h by flow cytometry.

**Figure 5:**
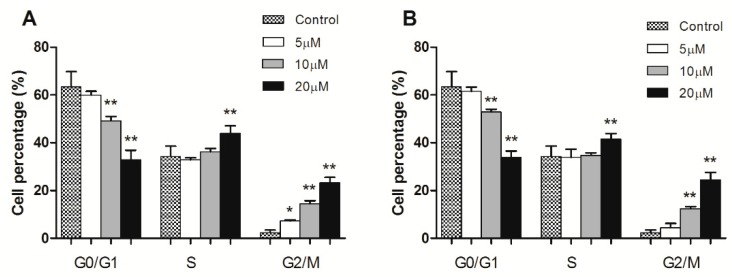
Histograms (**A**,**B**) showed the percentage of compounds **3** and **4** treated cells in different phases of the cell cycle, respectively. The values are expressed as mean ± SD of triplicate experiments. * *p* < 0.05, ** *p* < 0.01 compared with the control group.

To determine whether compound **3** or **4** affect different stages of the cell cycle, HeLa cells were treated with various concentrations (5 μM, 10 μM, 20 μM) of compounds **3** and **4**. Then cells were stained with propidium iodide (PI) and analyzed with flow cytometry. The accumulation of cells at the G2/M phase increased by 7.12% (5 μM), 15.45% (10 μM), and 22.97% (20 μM) at the treatment of compound **3**, dose-dependently (Figure 4). The number of cells treated with compound **4** at the G2/M phase increased by 4.88% (5 μM), 11.71% (10 μM), and 24.51% (20 μM), respectively, in a dose-dependent manner (Figure 4). While the proportion of cells in the G0/G1 phase significantly decreased as the concentrations of compounds **3** and **4** grew (*p* < 0.01) (Figure 5). Taken together, these results revealed that compounds **3** and **4** could inhibit HeLa cell proliferation through inducing G2/M phase arrest.

### 2.5. Apoptosis Detection

**Figure 6:**
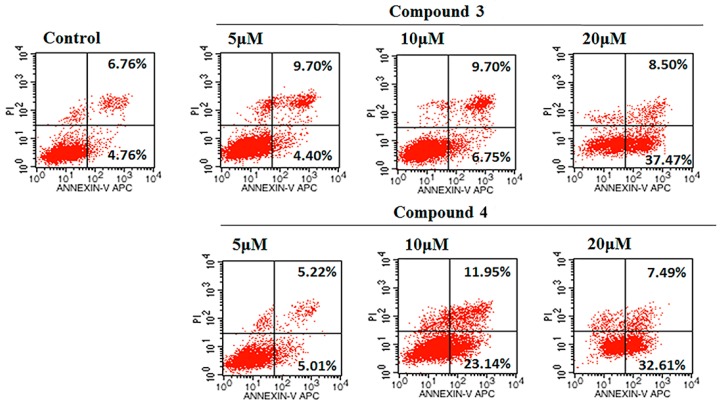
Apoptosis was assessed using Annexin V- fluorescein isothiocyanate (FITC) and propidium iodide (PI) staining after HeLa cells were treated with compounds **3** and **4** (5 μM, 10 μM, and 20 μM) for 24 h, measured by flow cytometry.

**Figure 7:**
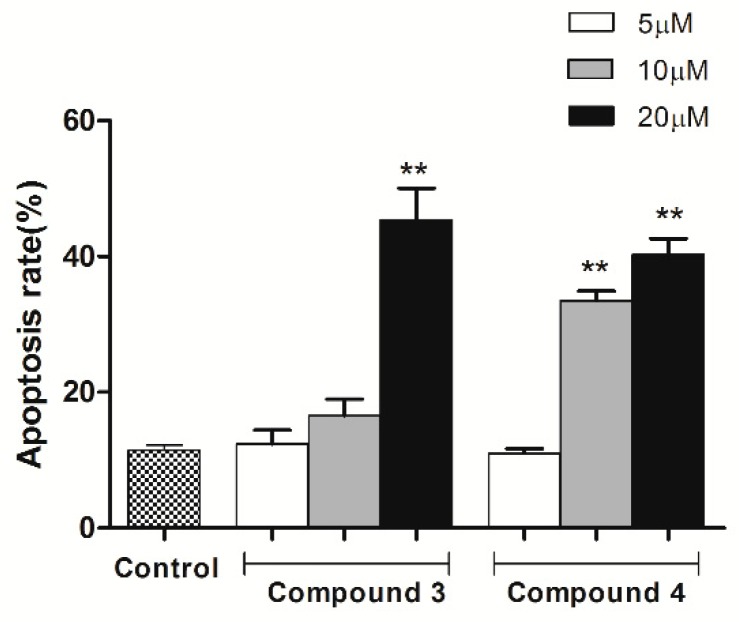
Histograms showed the apoptosis rate (early and late apoptosis) of HeLa cells treated with compounds **3** and **4**. The values are expressed as mean ± SD of triplicate experiments. ** *p* < 0.01 compared with the control group.

To confirm whether compound **3** or **4** inhibit cell viability through induction of apoptotic effects, HeLa cells treated with compound **3** or **4** were stained with Annexin V/PI and analyzed by flow cytometry. As shown in Figure 6, after treated with compound **3**, the apoptosis rate dose-dependently elevated from 11.52% to 14.1% (5 μM), 16.45% (10 μM), and 45.97% (20 μM) in HeLa cells, respectively. The proportion of apoptosis cells in compound **4** treated group exhibited a dose-dependently increase with 10.23% (5 μM), 35.09% (10 μM), and 40.10% (20 μM) in HeLa cells, respectively. Thus, treatment with compound **3** or **4** could significantly enhance the apoptosis (*p* < 0.01) in a dose-dependent manner (Figure 7).

### 2.6. Western Blot Analysis

**Figure 8:**
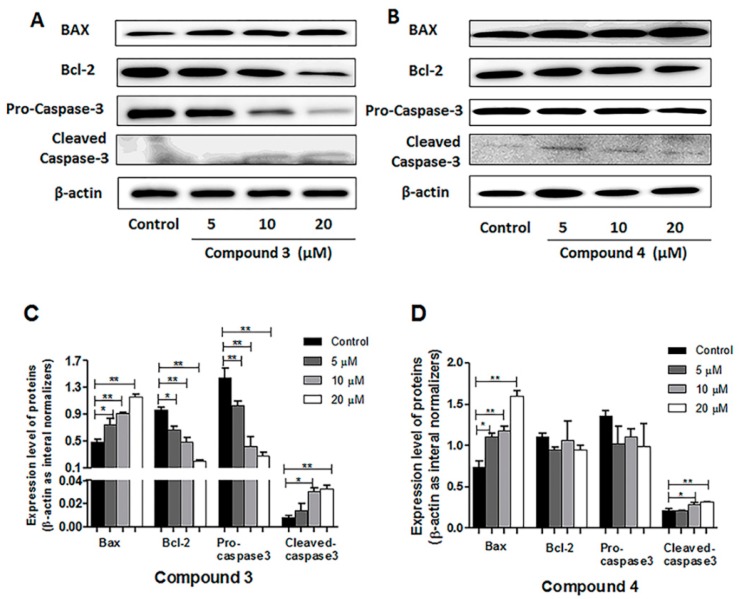
Western blots were performed to analyze relative protein expression levels involved in the cell apoptosis including Bax, Bcl-2, pro-caspase-3, and cleaved caspase-3(**A**,**B**). β -actin was used as a control. HeLa cells were treated with different concentrations (5, 10, 20 μM) of compound **3** or **4** for 24 h. The obvious change could be seen in the histogram in a concentration-dependent manner (**C**,**D**), * *p* < 0.05, ** *p* < 0.01, compared with the control group.

Following different concentrations of compounds **3** treatment, there was a decrease in the apoptosis-associated proteins, Bcl-2 and pro-caspase-3, while Bax and cleaved caspase-3 increased (Figure 8A). After treated with compound **4**, the pro-apoptotic protein Bax and cleaved caspase-3 were upregulated (Figure 8B). The ration of Bax/Bcl-2 after compound **4** treatment increased overall but not in a dose-dependent manner. The obvious change could be seen in the histogram in a concentration-dependent manner (Figure 8C,D, * *p* < 0.05, ** *p* < 0.01).

## 3. Discussion

Biflavonoids are the subclass of the flavonoid family with the flavonoid-flavonoid dimers structure. They are mainly distributed in gymnosperms [24] and have diverse pharmacological activities including anti-inflammatory activity, anticancer, antimicrobial, antiviral, and analgesic activity [25]. In our present study, we isolated and identified five amentoflavone-type biflavonoids from *G. biloba* flowers including a novel one, and then evaluated their activities of anti-proliferation in cancer cells.

It is now becoming clear that inflammation has an intimate connection with the occurrence and progression of tumors [26]. Anti-inflammatory therapy is efficacious for tumor prevention and suppression to some degree [26]. In our previous study, we evaluated the anti-inflammatory activities of the four known biflavonoids and found that bilobetin (**3**) and isoginkgetin (**4**) showed promising anti-inflammatory activities [20]. Bilobetin (**3**), one of biflavonoids, possesses a variety of biological activities [27,28,29]. However, the promising cytotoxic activity of bilobetin against cancer cells has not yet been well investigated . Isoginkgetin (**4**) was reported as a general inhibitor of pre-mRNA splicing [30] and can inhibit HT1080 tumor cell invasion by regulating PI3K/AKT1-dependent matrix metalloproteinase (MMP)-9 expression [18]. The anti-proliferation activities of both compounds **3** and **4** on the eight cancer cell lines in our study have never been previously reported. So, in our present study, we assessed the cytotoxic activities of the five biflavonoids including a novel one on different human cancer cells. Among them, we found that bilobetin (**3**) and isoginkgetin (**4**) exhibited better anti-proliferative activities in dose-dependent and time-dependent manners and Hela cells were more sensitive to compounds **3** and **4** than seven other cancer cells. Through Giemsa staining, the morphological structures of HeLa cells with compounds **3** and **4** altered significantly in a concentration-dependent manner, which was consistent with the detection of cell viability. Therefore, our study demonstrated that the potent anti-inflammatory compounds **3** and **4** also possess efficacious anticancer activities.

Since in our present study, compounds **3** and **4** with methoxyl groups at 4′ or/and 4′′′ in the B rings exhibited better anti-proliferative activities. So, the methoxyl group at 4′ and 4′′′ in the B rings of amentoflavone-type biflavonoids may be significant for their anti-proliferative activities, which are consistent with their better anti-inflammatory activities [20].

To further verify the underlying anti-proliferative mechanism of compounds **3** and **4**, their effects on cell cycle regulation and apoptosis were detected by flow cytometry. Since the cell cycle regulation is of vital importance in cancers, researchers have given sufficient weight to the control of the cell cycle [31]. Vanzyl et al. [32] have reported that isoginkgetin treatment arrested the G1, S, and G2 phases of the cell cycle in HCT116 colon cancer cells and A2780 ovarian cancer cells. In our study, we found that both compounds **3** and **4** could arrest the G2/M phase of the cell cycle in HeLa cells, which contributed to the inhibition of cell proliferation. Our results also indicate the disruption of pre-mRNA splicing is bound up with the cell cycle regulation in different phases as the function of isoginkgetin (**4**). Inducing cancer cell-specific apoptosis is considered to facilitate to treat cancers [4]. In our study, both compounds **3** and **4** could significantly elevate the proportion of apoptosis cells (*p* < 0.01) in a dose-dependent manner. To further investigate the mechanism of apoptosis induced by compounds **3** and **4**, western blot was performed to analyze the relative protein expression levels involved in the cell apoptosis. Bcl-2, a survival factor in the regulation of apoptosis and Bax—a proapoptotic regulator—were identified as key proteins of cell death and survival. They can promote the release of inner membrane space proteins such as cytochrome C. Then cytochrome C subsequently activate the caspase family and lead to the apoptotic cell death. Caspase-3 is a critical executioner in the downstream signal pathway of apoptosis. When activated, caspase-3 become cleaved caspase-3 [4]. Our results showed that compound **3** treatment visibly downregulated the level of antiapoptotic protein Bcl-2 and increased proapoptotic Bax. Supportively, the activated caspase-3 was upregulated. In response to the treatment of compound **4**, the expression of proapoptotic protein Bax was enhanced and caspase-3 was activated. Bcl-2 family proteins, including proapoptotic proteins, like Bax, Bad, Bim, and anti-apoptotic proteins, like Bcl-2, Bcl-X_L_, Bcl-W, are of vital importance in the mitochondrial pathway of cell apoptosis [33]. And there are many other proteins taking park in the regulation of cell apoptosis, which are not insular but interactional [34]. It is the balance between the pro- and anti-apoptotic proteins rather than the absolute quantity that determines the direction of apoptosis [33]. Though compound **4** did not demonstrate downregulation of Bcl-2, the ratio of Bax/Bcl-2 elevated, which might be the reason for induction of apoptosis after compound **4** treatment. However, the question of whether compound **4** can regulate other proteins to induce apoptosis still needs further and detailed investigation.

## 4. Materials and Methods 

### 4.1. General Experimental Procedures 

Agilent-NMR-vnmrs 600 spectrometers (Agilent Technologies Inc., Santa Clara, CA, USA) were used to measure the nuclear magnetic resonance (NMR) spectra with tetramethylsilane (TMS) as an internal standard. HR-ESI-MS were recorded on Agilent 6200 Series TOF and 6500 Series Q-TOF LC/MS System (Agilent Technologies, Inc., Santa Clara, CA, USA). Infrared spectra (IR) spectra were recorded by a Nicolet 5700 FT-IR microscope transmission (Thermo Scientific, Madison, WI, USA). TLC was carried out using silica gel 60 (400–600 mesh, Qingdao Marine Chemical Group Co., Qingdao, China). Column chromatography was carried out on Silica gel (200–300 mesh, Qingdao Marine Chemical Group Co. China), Sephadex LH-20 (Pharmacia, Uppsala, Sweden). 

EtOH, petroleum ether (PE), CHCl_3_, EtOAc, and n-BuOH (analysis-grade solvents) were obtained from Sinopharm Chemical Reagent Co., Ltd. (Hushi, Shanghai, China).

### 4.2. Plant Material

The fresh male flowers of *G. Biloba* were picked in Tancheng, Shandong Province of China in April 2015, and confirmed by Professor. Bin Li (Beijing Institute of Radiation Medicine, China). A voucher specimen (No. GBF150416) has been deposited in the Herbarium of Beijing Institute of Radiation Medicine.

### 4.3. Extraction and Isolation

The air-dried male flowers of *G. Biloba* (30 kg) were extracted four times with 70% EtOH aqueous solution. The ethanol extract (10kg) was then suspended in distilled water and partitioned successively with PE, CHCl_3_, EtOAc, and n-BuOH. The CHCl_3_ (200 g) and EtOAc (195 g) were subjected to silica gel column chromatography with elution using a stepwise gradient mixture of CHCl_3_ / MeOH (100:0→1:1) to yield 24 fractions (A-X) and 9 fractions (A-I) separately. The fraction I of EtOAc-soluble fraction were chromatographed on Sephadex LH-20 to obtain the subfractions E, which was further fractionated using a silica gel column with gradient elution of CHCl_3_ / MeOH (100:0→1:1) yielded compound **1** (6 mg). Compounds **3** (8 mg) and **5** (5 mg) were obtained from the CHCl_3_-soluble fraction. Compounds **2** (16 mg) and **4** (5 mg) were isolated from the EtOAc-soluble fraction. And the detailed isolation was presented in our previous study [20].

Compound (**1**): IR *ν*_max_ 3346, 2923, 1651, 1603, 1073, 833 cm^−1^. HR-ESI-MS *m/z* 701.1505 [M + H]^+^ (calcd for C_36_H_29_O_15_ ,701.1506). ^1^H- and ^13^C-NMR: see Table 2. The detailed infrared spectrum, HR-ESI-MS spectrum and NMR spectra can be found in the Supplementary Materials.

### 4.4. Cytotoxicity Assays 

The cytotoxic activity evaluation was carried on using the MTT assay [35]. The cells were seeded in 96-well plates at a density of 5 × 10^3^/well and incubated overnight at 37 °C and 5% CO_2_. Then cells were treated with prepared samples at concentrations from 3.1 μM to 100 μM for 48 h. For the time-dependent evaluation, Hela cells were incubated with the same concentrations of compound **3** or **4** for 24, 48, and 72 h, respectively. Cisplatin (Sigma, Life Science, St. Louis, MO, USA) was used as a positive control. After the different incubation time, 10 μL MTT reagents (VWR Life Science, AMRESCO LLC, Solon, OH, USA) were added to each well. Four hours later, the supernatant was removed carefully and 100 μL DMSO (Inno-chem, ACROS, Beijing, China) was added to each well. Absorbance was recorded at 540 nm using a microplate reader (Multiskan MK-3, Thermo, Waltham, MA, USA).

### 4.5. Cell Morphology Assay

Human cervical carcinoma cells were seeded in 24-well plates at a density of 1 × 10^4^ cells/well. After being treated with compound **3** or **4** for 48 h, cells were fixed with 4% paraformaldehyde for 30 min at room temperature. Subsequently, cells were washed with PBS and stained with Giemsa (Solarbio, Beijing, China) according to the manufacturer’s instructions. Morphological changes of the stained cells were observed and photographed under a fluorescence microscope (Nikon, ECLIPSE E600, Tokyo, Japan).

### 4.6. Cell Cycle Analysis

HeLa cells were treated with compound **3** or **4** at different concentrations. After incubating at 37 °C and 5% CO_2_ for 24 h, cells were trypsinized and washed twice with ice-chilled PBS. Then they were fixed in ice-cold 70% ethanol overnight at −20 °C. The fixed cells were treated with RNase A and propidium iodide (PI) (Beyotime, Nantong, China) and the DNA content was analyzed using a FACS Caliber (BD Biosciences, San Jose, CA, USA). Finally, flow cytometry analysis was accomplished by the Modifit software package (BD, Franklin Lakes, NJ, USA).

### 4.7. Apoptosis Detection

HeLa cells were treated with different concentrations of compound **3** or **4** for 24 h. The HeLa cells were harvested and washed. Staining of Annexin-V was done by using the Annexin V Apoptosis Kit (Beyotime, Nantong, China) according to the manufacturer’s instructions. After incubation at room temperature for 20 min in the dark, cells were stained by PI and immediately detected by flow cytometry (BD Biosciences, San Jose, CA, USA).

### 4.8. Western Blot Analysis

HeLa cells were washed twice with ice-cold PBS, and then protein was extracted from cells using RIPA buffer containing Protease Inhibitor Cocktail (MedChemExpress, Princeton, NJ, USA) on ice. After being quantified, protein samples were loaded and separated by 10% sodium dodecylsulfate polyacrylamide gel electrophoresis (SDS-PAGE). Then they were transferred to a polyvinylidene fluoride (PVDF) membrane. The PVDF membrane was blotted at room temperature for 3 h with 5% milk in Tris-buffered saline and Tween 20 (TBS-T). The membranes were incubated with diluted primary antibodies (1:1000 v/v, Cell Signaling Technology Inc., Beverly, MA, USA) overnight at 4 °C and subsequently incubated with diluted secondary antibodies (1:5000 v/v, Cell Signaling Technology Inc., Beverly, MA, USA) for 1 hour. Band signals were detected using enhanced chemiluminescence (ECL) detection system (Dakewe Biotech Co., Ltd., Shenzhen, China).

### 4.9. Statistical Analysis

Data were represented as mean ± SD. Statistical analysis was analyzed by GraphPad Prism 5 software using a one-way analysis of variance (ANOVA), followed by the Newman-Keuls Multiple Comparison Test. Differences with *p*-value < 0.05 were considered statistically significant and *p* < 0.01 was considered highly significant.

## 5. Conclusions

In our study, we isolated and identified a novel biflavonoid, amentoflavone 7″-*O*-β-d-glucopyranoside (**1**), from the male flowers of *Ginkgo*. This study evaluated the anti-proliferative activities of five biflavonoids on different cancer lines. Compounds **3** and **4** were screened out as the active compounds and we found HeLa cell was the most sensitive cell line. Compounds **3** and **4** exhibited considerable inhibitory activities on the proliferation of HeLa cells in a dose and time-dependent manner. The treatment of them also significantly altered the morphological structures dose-dependently. Besides, compounds **3** and **4** could arrest G2/M phase of cell cycle and induce cell apoptosis. Both of them could activate the proapoptotic protein Bax and the executor caspase-3. Compound **3** could also inhibit the antiapoptotic protein Bcl-2. Altogether, the investigation suggests compounds **3** and **4** might be considered as early lead compounds for anti-cancer drug discovery.

## Figures and Tables

**Table 1:** Cytotoxic activity of compound **1**–**5** against different cancer cell lines in vitro.

IC_50_ (μM)	Compound					
	1	2	3	4	5	Cisplatin
HepG2 ^a^	NE	NE	NE	34.00 ± 1.17	NE	9.19 ± 0.99
HeLa ^b^	NE	NE	14.79 ± 0.64	8.38 ± 0.63	NE	8.12 ± 0.23
NCI-H460 ^c^	NE	NE	36.42 ± 3.39	42.95 ± 2.14	NE	7.14 ± 0.14
Daudi ^d^	/	/	34.66 ± 3.78	20.07 ± 1.13	/	/
K562 ^e^	/	/	45.99 ± 0.27	18.76 ± 1.21	/	/
SKOV3 ^f^	/	/	79.19 ± 3.55	42.11 ± 2.00	/	/
MIAPaca-2 ^g^	/	/	97.28 ± 4.22	69.81 ± 5.17	/	/
MCF-7 ^h^	/	/	57.62 ± 2.11	91.19 ± 0.5	/	/

Data are described as means ± SD, *n* = 3; NE, no effect in 6.25-100 μM concentration range of compounds; **/**, undetected; ^a^ Human hepatocellular carcinoma cell line (HepG2). ^b^ Human cervical carcinoma cell line (HeLa). ^c^ Human large cell lung cancer cell line (NCI-H460). ^d^ Human lymphoma cell line (Daudi). ^e^ Human myelogenous leukemia cell line (K562). ^f^ Human ovarian adenocarcinoma cell line (SKOV3). ^g^ Human pancreatic carcinoma cell line (MIAPaca-2). ^h^ Human breast carcinoma cell line (MCF-7).

**Table 2:** ^1^H-NMR (600 MHz) and ^13^C-NMR (151 MHz) of compound **1** in DMSO-*d*_6_ (δ in ppm, *J* in Hz).

No.	δ_C_	δ_H_ (*J* in Hz)	No.	δ_C_	δ_H_ (*J* in Hz)
2	164.27		6′′	98.97	6.79 (d, *J* = 1.5,1H)
3	103.42	6.79 (d, *J* = 1.5,1H)	7′′	160.45	
4	182.13		8′′	106.69	
5	161.86		9′′	154.18	
6	99.32	6.18 (d, *J* = 2.0, 1H)	10′′	105.63	
7	164.57		1′′′	121.64	
8	94.45	6.45 (d, *J* = 2.0, 1H)	2′′′	128.87	7.6 (d, *J* = 8.8, 1H)
9	157.87		3′′′	116.23	6.72(d, *J* = 8.8, 1H)
10	104.12		4′′′	161.67	
1′	121.64		5′′′	116.23	6.72 (d, *J* = 8.8, 1H)
2′	128.27	8.01 (m, 1H)	6′′′	128.87	7.6 (d, *J* = 8.8, 1H)
3′	119.8		7′′-O-Glc		
4′	159.54		1′′′′	100.41	5.18 (d, *J* = 7.7, 1H)
5′	116.81	7.71 (d, *J* = 8.5, 1H)	2′′′′	73.48	3.08 (m,1H)
6′	132.15	8.01 (m, 1H)	3′′′′	77.81	3.42 (m,1H)
2′′	164.61		4′′′′	70.18	3.08 (m,1H)
3′′	103.23	6.87 (s, 1H)	5′′′′	76.77	3.26 (m,1H)
4′′	182.81		6′′′′	61.25	3.52 (m,1H)
5′′	161.23				3.72 (m,1H)

## Supplementary Materials

The following are available online, Figure S1: ^1^H-NMR spectrum of Amentoflavone 7′′-*O*-β-d-glucopyranoside (1) in DMSO-*d_6_*, Figure S2: ^13^C-NMR spectrum of Amentoflavone 7′′-*O*-β-d-glucopyranoside (1) in DMSO-*d_6_*, Figure S3: HSQC spectrum of Amentoflavone 7′′-*O*-β-d-glucopyranoside (1) in DMSO-*d_6_*, Figure S4: HMBC spectrum of Amentoflavone 7′′-*O*-β-d-glucopyranoside (1) in DMSO-*d_6_*, Figure S5: H^1^-H^1^ COSY spectrum of Amentoflavone 7′′-*O*-β-d-glucopyranoside (1) in DMSO-*d_6_*, Figure S6: NOESY spectrum of Amentoflavone 7′′-*O*-β-d-glucopyranoside (1) in DMSO-*d_6_*, Figure S7: HR-ESI-MS spectrum of Amentoflavone 7′′-*O*-β-d-glucopyranoside (1), Figure S8: IR spectrum of Amentoflavone 7′′-*O*-β-d-glucopyranoside (1).

## References

[1] Ni L., Zhao H., Tao L., Li X., Zhou Z., Sun Y., Chen C., Wei D., Liu Y., Diao G. (2018). Synthesis, in vitro cytotoxicity, and structure-activity relationships (SAR) of multidentate oxidovanadium(iv) complexes as anticancer agents. Dalton Trans..

[2] Du H., Huang Y., Hou X., Quan X., Jiang J., Wei X., Liu Y., Li H., Wang P., Zhan M. (2018). Two novel camptothecin derivatives inhibit colorectal cancer proliferation via induction of cell cycle arrest and apoptosis in vitro and in vivo. Eur J Pharm Sci..

[3] Ahmad K., Hafeez Z.B., Bhat A.R., Rizvi M.A., Thakura S.C., Azam A., Athara F. (2018). Antioxidant and apoptotic effects of *Callistemon lanceolatus* leaves and their compounds against human cancer cells. Biomed. Pharmacother..

[4] Mignani S., Rodrigues J., Tomas H., Zablocka M., Shi X.Y., Caminade A., Majoral J.P. (2018). Dendrimers in combination with natural products and analogues as anti-cancer agents. Chem. Soc. Rev..

[5] Zhang B., Huang R.Z., Hua J., Liang H., Pan Y.M., Dai L.M., Liang D., Wang H.S. (2016). Antitumor lignanamides from the aerial parts of *Corydalis saxicola*. Phytomedicine.

[6] Wang J.J. (2017). Ophiopogonin D’ Inhibits Prostate Cancer Cell Growth *in vitro* and *in vivo*, through Activating the RIP1/MLKL Pathway. Master’s Thesis.

[7] Youshikawa T., Naito Y., Kondo M. (1999). *Ginkgo Biloba* Leaf Extract–Review of Biological Actions. Antioxid. Redox. Signal..

[8] Mohanta T.K., Tamboli Y., Zubaidha P.K. (2014). Phytochemical and medicinal importance of *Ginkgo biloba* L.. Nat. Prod. Res..

[9] Zuo W., Yan F., Zhang B., Li J., Mei D. (2017). Advances in the Studies of *Ginkgo Biloba* Leaves Extract on Aging-Related Diseases. Aging Dis..

[10] Koch E. (2005). Inhibition of platelet activating factor (PAF)-induced aggregation of human thrombocytes by ginkgolides: Considerations on possible bleeding complications after oral intake of *Ginkgo biloba* extracts. Phytomedicine.

[11] Avula B., Sagi S., Gafner S., Upton R., Wang Y.H., Wang M., Khan I.A. (2015). Identification of *Ginkgo biloba* supplements adulteration using high performance thin layer chromatography and ultra high performance liquid chromatography-diode array detector-quadrupole time of flight-mass spectrometry. Anal. Bioanal. Chem..

[12] Ahmeda H.H., El-Abharb H.S., Hassaninc E.A.K., Abdelkaderb N.F., Shalabyc M.B. (2017). *Ginkgo biloba* L. leaf extract offers multiple mechanisms in bridling N-methylnitrosourea–mediated experimental colorectal cancer. Biomed. Pharmacother.

[13] Cao C., Su Y., Gao Y., Luo C., Yin L., Zhao Y., Chen H., Xu A. (2018). *Ginkgo biloba* Exocarp Extract Inhibits the Metastasis of B16-F10 Melanoma Involving PI3K/Akt/NF-kappaB/MMP-9 Signaling Pathway. Evid Based Complement. Alternat. Med..

[14] Liu Y.X., Yang B., Zhang L.R., Cong X.L., Liu Z., Hu Y., Zhang J., Hu H.X. (2018). Ginkgolic acid induces interplay between apoptosis and autophagy regulated by ROS generation in colon cancer. Biochem. Biophys. Res. Commun..

[15] Zhi Y., Pan J., Shen W., He P., Zheng J., Zhou X., Lu G., Chen Z., Zhou Z. (2016). Ginkgolide B Inhibits Human Bladder Cancer Cell Migration and Invasion Through MicroRNA-223-3p. Cell Physiol. Biochem..

[16] Yen T.H., Hsieh C.L., Liu T.T., Huang C.S., Chen Y.C., Chuang Y.C., Lin S.S., Hsu F.T. (2018). Amentoflavone Induces Apoptosis and Inhibits NF-kB-modulated Anti-apoptotic Signaling in Glioblastoma Cells. In Vivo.

[17] Pan L.L., Wu W.J., Zheng G.F., Han X.Y., He J.S., Cai Z. (2017). Ginkgetin inhibits proliferation of human leukemia cells via the TNF-α signaling pathway. Zeitschrift für Naturforschung C.

[18] Yoon S.O., Shin S., Lee H.J., Chun H.K., Chung A.S. (2006). Isoginkgetin inhibits tumor cell invasion by regulating phosphatidylinositol 3-kinase/Akt-dependent matrix metalloproteinase-9 expression. Mol. Cancer Ther..

[19] Wang G.X., Yang Y.Z., Cao F.L., Zhang X.Y. (2013). Studies on the Biological Character of Flowering of Ancient Male *Ginkgo biloba* Trees in Different Areas 1: Analysis of Flowering Phenology and the Feature of Flower Spikes. Chinese Ag. Sci. Bullet..

[20] Li M., Li B., Hou Y., Tian Y., Chen L., Liu S.J., Zhang N., Dong J.X. (2019). Anti-inflammatory Effects of Chemical Components from *Ginkgo biloba* L. Male Flowers on Lipopolysaccharide-Stimulated RAW264.7 Macrophages. Phtother. Res..

[21] Markham K.R., Sheppard C., Hans G. (1987). ^1^H,^13^C-NMR studies of some naturally occurring amentoflavone and hinokiflavone biflavone. Phytochemistry.

[22] Dora G., Edwards J.M. (1991). Taxonomic status of *Lanaria lanata* and isolation of a novel biflavone. J. Nat. Prod..

[23] Zhang N., Lu J.C., Wang J., Han L., Yang X.G. (2009). Isolation and identification of chemical constituents from the needles of *Taxusmedia*. J. Shenyang Pharm. Univ..

[24] Moawad A., Amir D. (2016). Ginkgetin or Isoginkgetin: The Dimethylamentoflavone of Dioon edule Lindl. Leaves. European J. Med. Plants.

[25] Kim H.P., Park H., Son K.H., Chang H.W., Kang S.S. (2008). Biochemical pharmacology of biflavonoids: Implications for anti-inflammatory action. Arch. Pharm. Res..

[26] Coussens L.M., Werb Z. (2002). Inflammation and cancer. Nature.

[27] Dell’Agli M., Galli G.A., Bosisio E. (2006). Inhibition of cGMP-Phosphodiesterase-5 by biflavones of *Ginkgo biloba*. Planta Med..

[28] Lee M.K., Lim S.W., Yang H., Sung S.H., Lee H.S., Parkc M.J., Kim Y.C. (2006). Osteoblast differentiation stimulating activity of biflavonoids from *Cephalotaxus koreana*. Bioorganic. Med. Chem. Lett..

[29] Krauze Baranowskaa M. (2003). and Wiwartb, M. Antifungal Activity of Biflavones from *Taxus baccata* and *Ginkgo biloba*. Zeitschrift fur Naturforschung C.

[30] O’Brien K., Matlin A.J., Lowell A.M., Moore M.J. (2008). The Biflavonoid Isoginkgetin Is a General Inhibitor of Pre-mRNA Splicing. J. Biol. Chem..

[31] Harashima H., Dissmeyer N., Schnittger A. (2013). Cell cycle control across the eukaryotic kingdom. Trends Cell Biol..

[32] Vanzyl E.J., Rick K.R.C., Blackmore A.B., MacFarlane E.M., McKay B.C. (2018). Flow cytometric analysis identifies changes in S and M phases as novel cell cycle alterations induced by the splicing inhibitor isoginkgetin. PLoS ONE.

[33] Wong R.S.Y. (2011). Apoptosis in cancer: From pathogenesis to treatment. J. Exper. Clinical Cancer Res..

[34] Karabay A.Z., Koc A., Ozkan T., Hekmatshoar Y., Sunguroglu A., Aktan F., Buyukbing Z. (2016). Methylsulfonylmethane Induces p53 Independent Apoptosis in HCT-116 Colon Cancer Cells. Int. J. Mol. Sci..

[35] Choudhury B., Kandimalla R., Elancheran R., Bharali R., Kotoky J. (2018). *Garcinia morella* fruit, a promising source of antioxidant and anti-inflammatory agents induces breast cancer cell death via triggering apoptotic pathway. Biomed. Pharmacother..

