# Supplementary material for: Anticancer Effects of Five Biflavonoids from Ginkgo Biloba L. Male Flowers In Vitro
Source: Molecules. 2019 Apr 16;24(8):1496. doi: 10.3390/molecules24081496 (PMC6514578; doi:10.3390/molecules24081496)
Supplement: Supplementary file 1 [file molecules-24-01496-s001.pdf]

Supporting information for

Anticancer Effects of Five Biflavonoids from *Ginkgo biloba* L. Male Flowers *in Vitro*

Min Li[a], Dan Zhang[a,b], Bin Li[a], Ziming Xia[a], Fengjun Xiao[a], Ying Tian[a], Wenjing Rui[a], Junxing Dong[*a]

[a] *Beijing Institute of Radiation Medicine, Beijing 100850, People's Republic of China*

[b] *Jilin University, Changchun130012, People's Republic of China*

Figure S1. ^{1}H-NMR spectrum of Amentoflavone 7′′-O-β-D-glucopyranoside (1) in DMSO-d_6

Figure S2. ^{13}C-NMR spectrum of Amentoflavone 7′′-O-β-D-glucopyranoside (1) in DMSO-d_6

Figure S3. HSQC spectrum of Amentoflavone 7′′-O-β-D-glucopyranoside (1) in DMSO-d_6

Figure S4. HMBC spectrum of Amentoflavone 7′′-O-β-D-glucopyranoside (1) in DMSO-d_6

Figure S5. H-H COSY spectrum of Amentoflavone 7′′-O-β-D-glucopyranoside (1) in DMSO-d_6

Figure S6. NOESY spectrum of Amentoflavone 7′′-O-β-D-glucopyranoside (1) in DMSO-d_6

Figure S7. HR-ESI-MS spectrum of Amentoflavone 7′′-O-β-D-glucopyranoside (1)

Figure S8. IR spectrum of Amentoflavone 7′′-O-β-D-glucopyranoside (1)

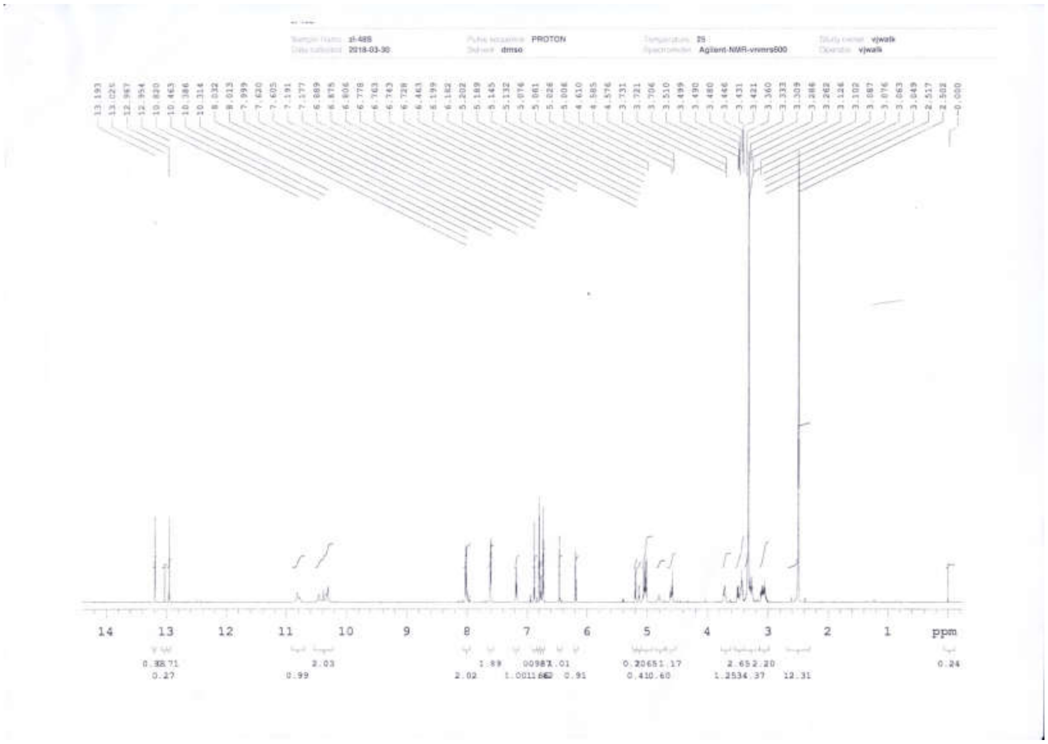

Figure S1. ^{1}H-NMR spectrum of Amentoflavone 7″-O-β-D-glucopyranoside (1) in DMSO-d_6

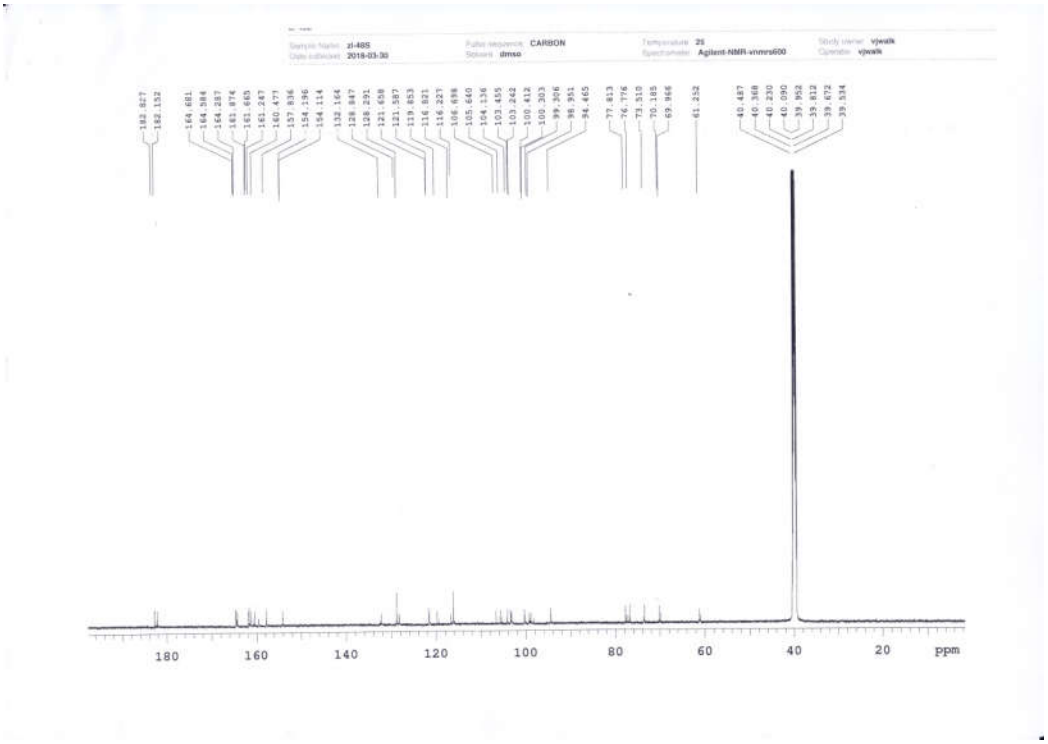

Figure S2. ^{13}C-NMR spectrum of Amentoflavone 7″-O-β-D-glucopyranoside (1) in DMSO-d_6

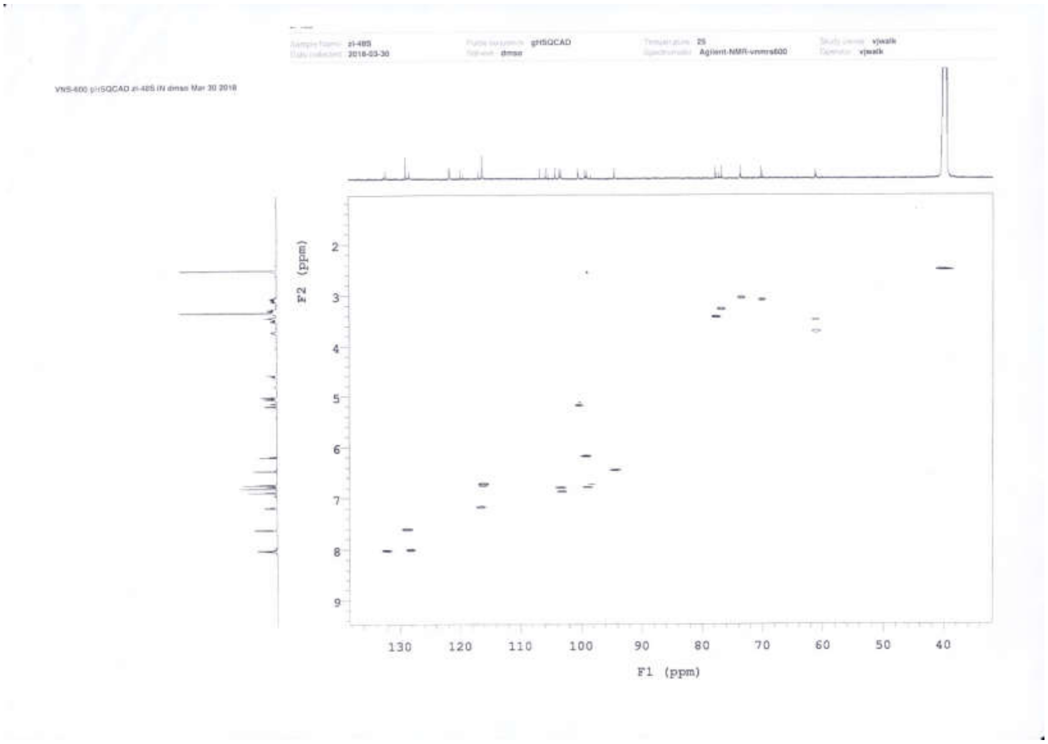

Figure S3. HSQC spectrum of Amentoflavone 7″-O-β-D-glucopyranoside (1) in DMSO-d_6

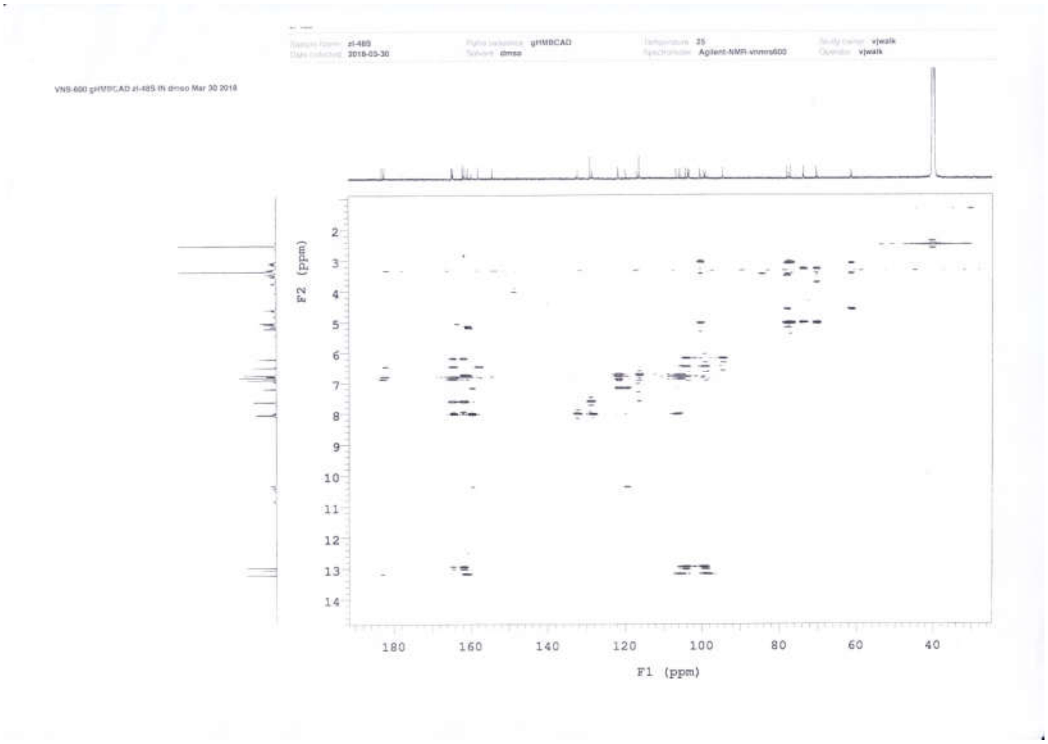

Figure S4. HMBC spectrum of Amentoflavone 7′′-O-β-D-glucopyranoside (1) in DMSO-d_6

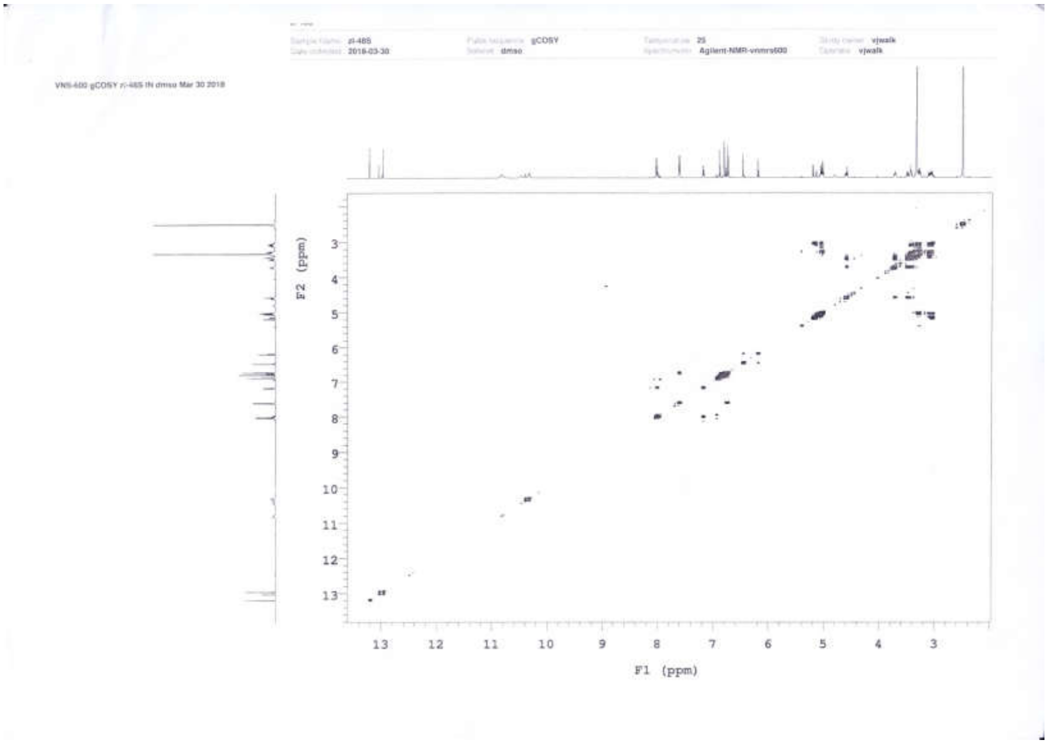

Figure S5. H-H COSY spectrum of Amentoflavone 7′′-O-β-D-glucopyranoside (1) in DMSO-d_6

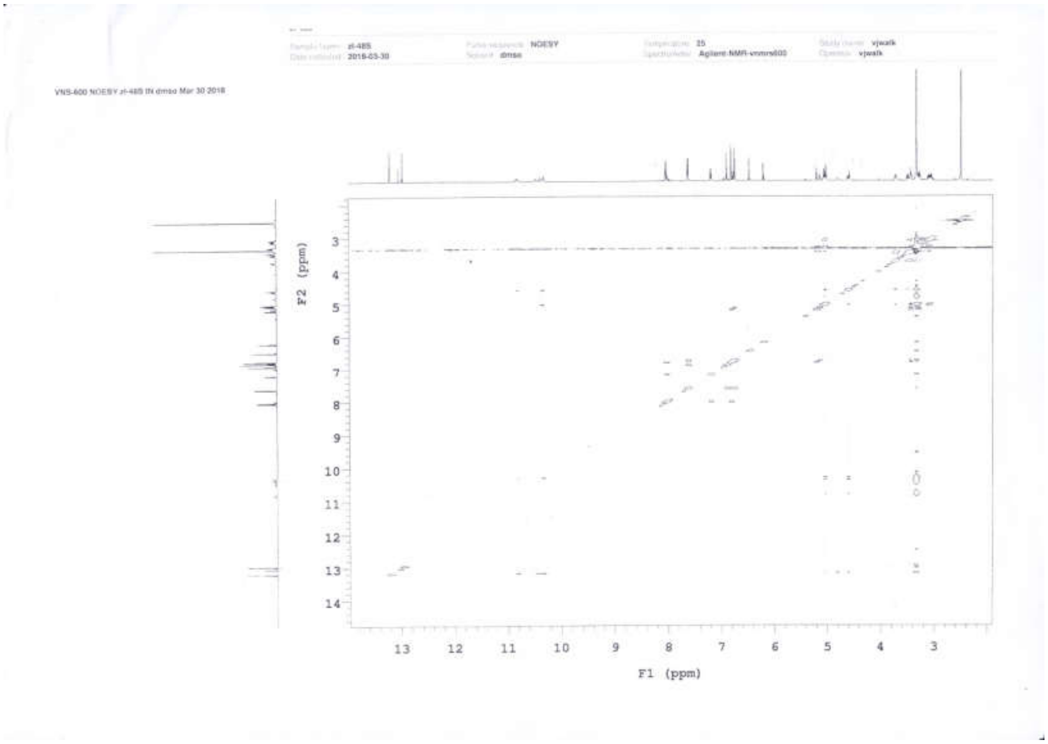

Figure S6. NOESY spectrum of Amentoflavone 7″-O-β-D-glucopyranoside (1) in DMSO-d_6

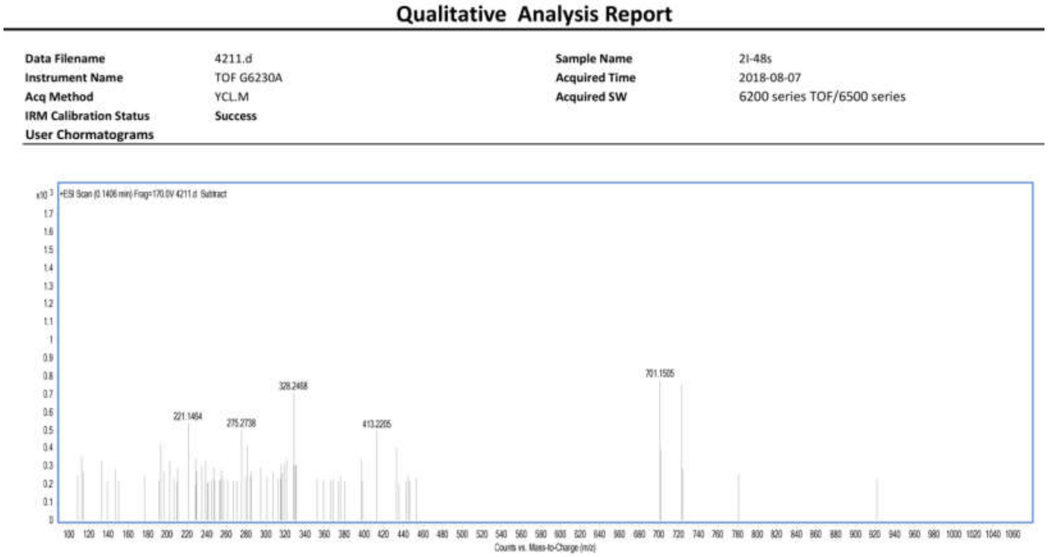

Figure S7. HR-ESI-MS spectrum of Amentoflavone 7″-O-β-D-glucopyranoside (1)

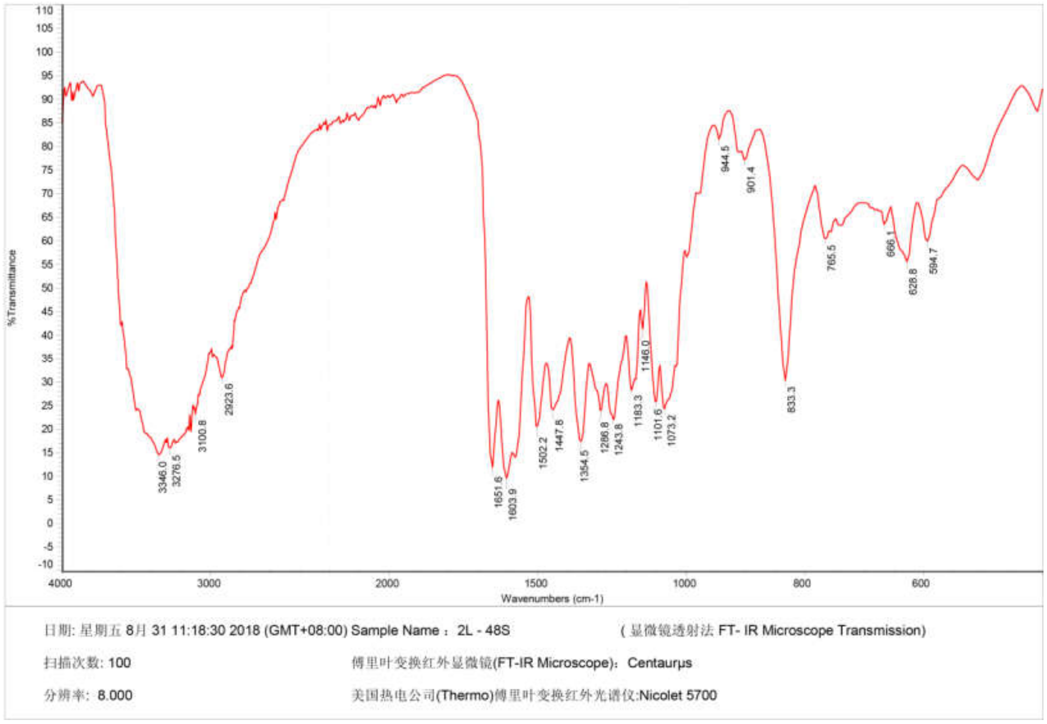

Figure S8. IR spectrum of Amentoflavone 7″-O-β-D-glucopyranoside (1)
